# Supplementary material for: Gomisin M2 Ameliorates Atopic Dermatitis-like Skin Lesions via Inhibition of STAT1 and NF-κB Activation in 2,4-Dinitrochlorobenzene/Dermatophagoides farinae Extract-Induced BALB/c Mice
Source: Molecules. 2021 Jul 21;26(15):4409. doi: 10.3390/molecules26154409 (PMC8346973; doi:10.3390/molecules26154409)
Supplement: Supplementary file 1 [file molecules-26-04409-s001.zip › supplementary Figure S1.pdf]

Gomisin M2 ameliorates atopic dermatitis-likes skin lesions via inhibition of STAT1 and NF-κB activation in 2, 4-dinitrochlorobenzene/ *Dermatophagoides farinae* extract-induced BALB/c mice

Jinjoo Kang[a,1], Soyoung Lee[b,1], Namkyung Kim[a], Hima Dhakal[a], Taeg Kyu Kwon[c], Eun-Nam Kim[d], Gil-Saeng Jeong[d,*], Sang-Hyun Kim[a,**]

[a] *Department of Pharmacology, School of Medicine, Kyungpook National University, Daegu, Republic of Korea*

[b] *Immunoregulatory Materials Research Center, Korea Research Institute of Bioscience and Biotechnology, Jeongeup, Republic of Korea*

[c] *Department of Immunology, School of Medicine, Keimyung University, Daegu, Republic of Korea*

[d] *College of Pharmacy, Keimyung University, Daegu, Republic of Korea*

[1] Equally contributed.

* Corresponding author. College of Pharmacy, Keimyung University, Daegu, Republic of

Korea.

** Corresponding author. Department of Pharmacology, School of Medicine, Kyungpook National University, Daegu, Republic of Korea.

E-mail addresses: gsjeong@kmu.ac.kr (G-S Jeong), shkim72@knu.ac.kr (S-H Kim).

Supplementary materials and methods

Measure of body weight

The body weight of each mouse was carefully monitored and measured using a HKC65050 (Cas, Incheon, Republic of Korea) after DNCB application during the study once weekly.

Figure S1. Effects of GM2 on the body weight of mice.

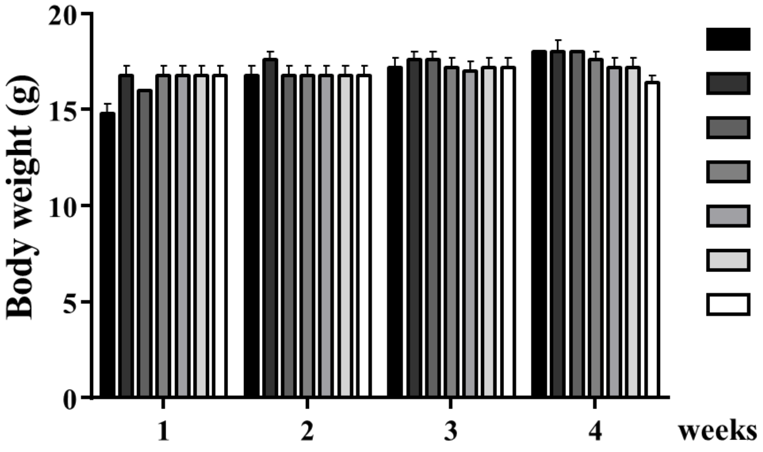

The body weights of the mice were measured on the first day of each week for 4 weeks using a HKC65050 electronic balance. Data are presented as a graph represents the means ± SEM (*n*= 5). Compared with the DNCB/DFE-induced group. Dexa: dexamethasone.
