# Supplementary material for: Gomisin M2 Ameliorates Atopic Dermatitis-like Skin Lesions via Inhibition of STAT1 and NF-κB Activation in 2,4-Dinitrochlorobenzene/Dermatophagoides farinae Extract-Induced BALB/c Mice
Source: Molecules. 2021 Jul 21;26(15):4409. doi: 10.3390/molecules26154409 (PMC8346973; doi:10.3390/molecules26154409)
Supplement: Supplementary file 1 [file molecules-26-04409-s001.zip › supplementary Figure S2.pdf]

Gomisin M2 ameliorates atopic dermatitis-likes skin lesions via inhibition of STAT1 and NF-κB activation in 2, 4-dinitrochlorobenzene/ *Dermatophagoides farinae* extract-induced BALB/c mice

Jinjoo Kang[a,1], Soyoung Lee[b,1], Namkyung Kim[a], Hima Dhakal[a], Taeg Kyu Kwon[c], Eun-Nam Kim[d], Gil-Saeng Jeong[d,*], Sang-Hyun Kim[a,**]

[a] *Department of Pharmacology, School of Medicine, Kyungpook National University, Daegu, Republic of Korea*

[b] *Immunoregulatory Materials Research Center, Korea Research Institute of Bioscience and Biotechnology, Jeongeup, Republic of Korea*

[c] *Department of Immunology, School of Medicine, Keimyung University, Daegu, Republic of Korea*

[d] *College of Pharmacy, Keimyung University, Daegu, Republic of Korea*

[1] Equally contributed.

* Corresponding author. College of Pharmacy, Keimyung University, Daegu, Republic of

Korea.

** Corresponding author. Department of Pharmacology, School of Medicine, Kyungpook National University, Daegu, Republic of Korea.

E-mail addresses: gsjeong@kmu.ac.kr (G-S Jeong), shkim72@knu.ac.kr (S-H Kim).

Supplementary materials and methods

Cell viability

The cell viability was determined using the 3-(4,5-dimethylthiazol-2-yl)-2,5-diphenyltetrazolium bromide (MTT) assay. Briefly, HaCaT cells (1×10^4 cells/ 96-well plate, n=5) were treated with the GM2 at different concentrations (0 - 100 μM) or DMSO (0.5%) for 24 h, followed by incubation with 20 μL of MTT solution (5 mg/mL) for 4 h. The formed formazan crystals were dissolved in dimethyl sulfoxide (99.5 %, 100 μL). The absorbance was determined using a plate reader at 570 nm (Molecular Devices, Sunnyvale, CA). The relative cell viability (%) was shown as a percentage compared to non-treated control cells (100 %).

Figure S2. Effects of GM2 on cell viability of keratinocytes.

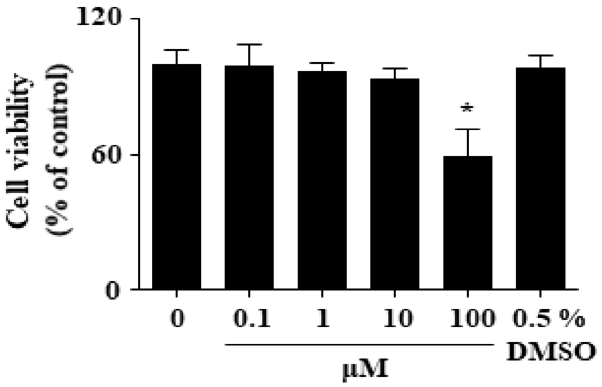

Effects of GM2 on cytotoxicity in keratinocytes. The viability of the HaCaT cells was measured using the MTT assay. Data are presented as a graph represents the means ± SEM (n= 5). *$p < 0.05$, compared with the non-treated control group.
