# Supplementary material for: Gomisin M2 Ameliorates Atopic Dermatitis-like Skin Lesions via Inhibition of STAT1 and NF-κB Activation in 2,4-Dinitrochlorobenzene/Dermatophagoides farinae Extract-Induced BALB/c Mice
Source: Molecules. 2021 Jul 21;26(15):4409. doi: 10.3390/molecules26154409 (PMC8346973; doi:10.3390/molecules26154409)
Supplement: Supplementary file 1 [file molecules-26-04409-s001.zip › supplementary Table S1.pdf]

Gomisin M2 ameliorates atopic dermatitis-likes skin lesions via inhibition of STAT1 and NF-κB activation in 2, 4-dinitrochlorobenzene/ *Dermatophagoides farinae* extract-induced BALB/c mice

Jinjoo Kang[a,1], Soyoung Lee[b,1], Namkyung Kim[a], Hima Dhakal[a], Taeg Kyu Kwon[c], Eun-Nam Kim[d], Gil-Saeng Jeong[d,*], Sang-Hyun Kim[a,**]

[a] *Department of Pharmacology, School of Medicine, Kyungpook National University, Daegu, Republic of Korea*

[b] *Immunoregulatory Materials Research Center, Korea Research Institute of Bioscience and Biotechnology, Jeongeup, Republic of Korea*

[c] *Department of Immunology, School of Medicine, Keimyung University, Daegu, Republic of Korea*

[d] *College of Pharmacy, Keimyung University, Daegu, Republic of Korea*

[1] Equally contributed.

* Corresponding author. College of Pharmacy, Keimyung University, Daegu, Republic of

Korea.

** Corresponding author. Department of Pharmacology, School of Medicine, Kyungpook National University, Daegu, Republic of Korea.

E-mail addresses: gsjeong@kmu.ac.kr (G-S Jeong), shkim72@knu.ac.kr (S-H Kim).

Table S1. Primer pair sequences used for qPCR, and the antibodies used for Western blot analysis.

		Human	
**Gene**	**F (5′→3′)**	**R (5′→3′)**	**Accession numbers**
CCL22	AGG ACA GAG CAT GGA TCG CCT ACA	TAA TGG CAG GGA GGT AGG GCT CCT	NM_002990.5
CXCL8	GGT GCA GTT TTG CCA AGG AG	TGC TTG AAG TTT CAC TGG CAT C	NM_000584.4
IL-1β	GCT GAT GGC CCT AAA CAG ATG AA	TGA AGC CCT TGC TGT AGT GGT G	NM_000576.3
IL-6	AAA GAG GCA CTG GCA GAA AA	ATC TGA GGT GCC CAT GCT AC	NM_000600.5
GAPDH	CGA CCA CTT TGT CAA GCT CA	AGG GGA GAT TCA GTG TGG TG	NM_001357943.2

		Mouse	
**Gene**	**F (5′→3′)**	**R (5′→3′)**	**Accession numbers**
TGF-β	TGA TAC GCC TGA GTG GCT GTC T	CAC AAG AGC AGT GAG CGC TGA A	NM_011577.2
IFN-γ	TCA AGT GGC ATA GAT GTG GAA GAA	TGG CTC TGC AGG ATT TTC ATG	NM_008337.4
IL-1β	ATA ACC TGC TGG TGT GTG AC	AGG TGC TGATGT ACC AGT TG	NM_008361.4
IL-4	TCG GCA TTT TGA ACG AGG TC	GAA AAG CCC GAA AGA GTC TC	NM_021283.2
IL-5	GAA GTG TGG CGA GGA GAG AC	GCA CAG TTT TGT GGG GTT TT	NM_010558.1
IL-6	TCC AGT TGC CTT CTT GGG AC	GGT CTG TTG GGA GTG GTA TC	NM_001314054.1
IL-12a	GAT GAC ATG GTG AAG ACG GC	AGG CAC AGG GTC ATC ATC AA	NM_001159424.2
IL-17A	TTT AAC TCC CTT GGC GCA AAA	CTT TCC CTC CGC ATT GAC AC	NM_010552.3
TSLP	CTC CCC TGC ACA CAC CTT AC	TCA GAC CAC CTC ATC ATG GC	NM_021367.2
β-actin	ACC CTA AGG CCA ACC GTG AA	ATG GCG TGA GGG AGA GCA TAG	NM_007393.5

IL, Interleukin; CCL, Chemokine (C-C motif) ligand; IFN, Interferon; GAPDH, Glyceraldehyde-3-phosphate dehydrogenase; CCR, C-C chemokine receptor; CXCL, C-X-C motif chemokine; TSLP, Thymic stromal lymphopoietin.

**Target protein**	**Supplier**	**Cat#**	**Size (kDa)**	**Host**	**Dilution**
CD4	Abcam, MA, USA	Ab25804	50, 54	Mouse	1:1000
CD44	Cell Signaling, MA, USA	#5640	81	Mouse	1:1000
IκBα	Cell Signaling, MA, USA	#9242	39	Rabbit	1:1000
β-actin (C4)	Santa cruz, Germany	SC-47778	43	Mouse	1:4000
NF-κB p65	Abcam, MA, USA	ab16502	64	Rabbit	1:1000
Lamin B1	Santa cruz, Germany	SC365962	67	Mouse	1:2000
phospho-STAT1^{Tyr701}	Cell Signaling, MA, USA	#9167	84, 91	Rabbit	1:2000
STAT1	Cell Signaling, MA, USA	#9172	84, 91	Rabbit	1:2000
